# The Poetical Works of the Late Richard S. Gedney, with a Memoir, Etc.

**Published:** 1858-01

**Authors:** James Ogden


### ARTICLE V.

*The Poetical Works of the late* Richard S. Gedney, with a Memoir, etc., by James Ogden, M. D., etc.

''It is too frequently the case that the poetry of the unknown dead which is published, is of that kind that would have been much better buried with them. Not so this volume. The author was an American boy, born in Ulster county in this state, sent to England at two years old, and there educated until the time of his death, a year ago, July 15, 1856, at Cheltenham, at the early age of seventeen. The entire volume shows not only a promise of greatness as a poet had the boy lived, but a maturity and a success already, which is not a little surprising. In fact, the book is little less than a miracle when the youth of the author is duly considered. He well says :

Through these pages there lie scattered
Gems whose worth few eyes may see,
Yet a vase, though small and shattered,
If God will, shall hold the sea.

Think the poet not assumptuous,
Howsoever few his years,
Even youth is not presumptuous
When its pride is blent with tears.

And remember, that the morning
Comes before the sun's full ray—
This is but the poet's dawning,
Judge not harshly of his day.

Just before his death he wrote

MY DIRGE.

Let the bell toll! another soul
Has passed the Stygian river;
Without a fear, without a tear,
'Twas render'd to the Giver.

To God's high throne that young heart's moan
"In pity, spare!" ascended—
Now, spared the woe that reigns below,
That mournful prayer is ended.

Sorrow and doom, and fear and gloom,
No more within its vision;
It now doth raise soft hymns of praise
In happiness Elysian!

Over the grave of such a boy not friends alone, but all who admire genius and honor the soul of a true poet, however young and inexperienced he was, do well to weep."

These remarks and quotations are copied from Harper's Weekly, of September 5, 1857.

The young poet so justly complimented and so full of genius, was the son of the widely known and justly distinguished Dr. E. Gedney, a native of the state of New York, formerly in dental practice in the city of New York, more recently in Manchester, England; and now at his beautiful country seat, Malvern Hall, opposite Hyde Park, on the Hudson.

Before leaving for England, in 1842, Dr. Gedney loaned his dental library, the most extensive, at that time, in America, to the American Society of Dental Surgeons, with the librarian of which, it still remains. He has amassed an abundant fortune by his profession, of which he has been a distinguished ornament; and will probably return to Europe to spend the remnant of his days, if he can dispose of his estate on the Hudson to his entire satisfaction.

Of the poetry of his departed son, published in an elegant octavo by the Appletons, the London Critic of July 15, 1857, thus speaks:

"Richard Solomon Gedney was the son of E. Gedney, Esq., of Malvern Hall, Ulster county, in the state of New York. He was born on the 15th of Oct., 1838. At the age of two years he was brought to England, and afterwards received the rudiments of a liberal education under Dr. Hodgson, of Chorlton High School, near Manchester. Subsequently, he was transferred to the College at Cheltenham, and here it was that the selfsame malady which had stricken Kirke White and Keats, came once again to extinguish all that was mortal in a youth of genius. This is almost all we know of Richard S. Gedney, save that he died on the 15th of July, 1856, after a protracted illness, in which the poet's love and the christian's faith brought him glimpses of beauty even to the last, and that his body was embalmed and forwarded to America. It is a melancholy history, brief as it is melancholy, not that a youth died at the age of seventeen, for that is not uncommon, but because a mind was recalled from earth, which might have done so much to brighten, and bless, and beautify it. It would not be generous—nay, it would not even be just to apply the same rigid rules of criticism to this volume as we should have applied had the author lived and published at the age of thirty. What can we expect but that much which is immature in composition should mark immaturity of years? And yet, if nine-tenths of the poets who have had the privilege of long life and long experience, had only written with half the power and pathos of this wonderful boy, we should have assigned them a rank in literature. We pause in absolute wonder over some of those pages, and frequently forget the erratic course of the author's imagination in its evident power.

We shall quote a few stanzas from a poem entitled "The Death Ride," which refers to the celebrated charge of the Light Brigade, at Balaklava, and we desire our readers to mark their character. There is a remarkable similarity between this poem and one by the poet laureate on the same subject. Now, as no one will dare to accuse Tennyson of imitation, it is but fair to the memory of Mr. Gedney, to show that he is equally free from such a charge, at least in this instance; for "The Death Ride" was published in the Manchester Weekly Advertiser the same week as the intelligence of the battle reached England, and, therefore, before Tennyson's celebrated poem appeared. This remarkable occurrence ought to make critics cautious. It confirms our previous opinion that the elder D'Israeli expended much labor and research to little purpose when he wrote his chapter on "Poetical Imitations and Similarities." The shrewd, but somewhat cumbrous compiler probably chased a shadow, since what he terms "imitations" and traces of design may after all, be merely accidental coincidences.

THE DEATH RIDE.

**On, o'er the rocky ground,**
**Cannon on all sides round,**
**Belching forth death and wound,**
**Madly they rode.**

**On! like a demon blast,**
**Thundering and fierce and fast,**
**Fear to the winds they cast,**
**Needing no goad.**

**On! through the rocky dell!**
**On! through the cannon's hell!**
**On! though by heaps they fell,**
**Dying and dead.**

**On! with a whirlwind's leap!**
**Down on the Russ they sweep!**
**Madly their swords they steep,**
**Where the foe bled.**

Only a few hours before his death, the young poet wrote his dirge. It will awaken mournful ideas, for it was the gifted boy's last effusion. Through the whole of Mr. Gedney's poems we trace the personal history of the writer. There is a mournful tone ringing like a knell through the pages, a feeling of regret, that the beautiful forms of earth must so soon pass away. But though there is sadness, there is nothing like despair ; it is only a natural and a manly grief. It is too painfully apparent that the affections had been aroused to that intensity which belongs exclusively to the poetic character aroused, and by unfortunate checks, stretched to the very verge of torture. We can almost see the radiant life throbbing itself away under the pressure of disappointments, which, whether fancied or real, are no less difficult to bear. The last throb is past, but this book remains to show how much beauty, and power, and sensibility had their empire in the brain of a mere boy."

The elegantly executed likeness of the young poet, which is inserted before the title page, manifests a remarkable maturity of physical, as well as of mental development in the subject of this memoir—and it is accompanied by a well written biography which imparts great interest to the volume.

This elegant ornament of the drawing-room table, can be procured of the Appletons, of New York, at $5 00, gilt edged, on thick paper, and bound superbly. It should be found in the library of every wealthy member of the dental profession, as well on account of the talents of the son, as of the reputation of the father.

